# Deposition of Hybrid Photocatalytic Layers for Air Purification Using Commercial TiO_2_ Powders

**DOI:** 10.3390/molecules26216584

**Published:** 2021-10-30

**Authors:** Ewoud Cosaert, Cédric Wolfs, Stéphanie D. Lambert, Geraldine J. Heynderickx, Dirk Poelman

**Affiliations:** 1 LumiLab, Department of Solid State Sciences, Ghent University, 9000 Ghent, Belgium; Ewoud.Cosaert@UGent.be; 2 Nanomaterials, Catalysis & Electrochemistry, Department of Chemical Engineering, University of Liège, 4000 Liège, Belgium; Cedric.Wolfs@ULiege.be (C.W.); Stephanie.Lambert@ULiege.be (S.D.L.); 3 Laboratory for Chemical Technology, Ghent University, 9000 Ghent, Belgium; Geraldine.Heynderickx@UGent.be

**Keywords:** photocatalysis, TiO_2_, sol–gel, PEG, PMMA, PVP, air purification

## Abstract

Photocatalytic nanomaterials, using only light as the source of excitation, have been developed for the breakdown of volatile organic compounds (VOCs) in air for a long time. It is a tough challenge to immobilize these powder photocatalysts and prevent their entrainment with the gas stream. Conventional methods for making stable films typically require expensive deposition equipment and only allow the deposition of very thin layers with limited photocatalytic performance. The present work presents an alternative approach, using the combination of commercially available photocatalytic nanopowders and a polymer or inorganic sol–gel-based matrix. Analysis of the photocatalytic degradation of ethanol was studied for these layers on metallic substrates, proving a difference in photocatalytic activity for different types of stable layers. The sol–gel-based ${\mathrm{TiO}}_{2}$ layers showed an improved photocatalytic activity of the nanomaterials compared with the polymer ${\mathrm{TiO}}_{2}$ layers. In addition, the used preparation methods require only a limited amount of photocatalyst, little equipment, and allow easy upscaling.

## 1. Introduction

It has been known for several decades that exposure to volatile organic compounds (VOCs) forms a risk for human health [1], including the so-called sick-building syndrome [2]. VOCs originating from building materials can cause headache, nausea, and irritation of the throat, but also can result in increased fatigue and neurological symptoms, such as concentration difficulty and depression. Some VOCs, such as benzene and formaldehyde, are even carcinogenic [3]. VOCs are emitted from paints, cleaning chemicals, furniture, textiles, etc. [4]. Since humans spend a lot of their time indoors, this is an important issue to solve.

Ever since the discovery of the photocatalytic water splitting property of titanium dioxide (${\mathrm{TiO}}_{2}$) electrodes by Fujishima and Honda in 1972 [5], this phenomenon has gained interest in many research fields, such as photo-oxidation/photodegradation of aqueous/gaseous pollutants, ${\mathrm{CO}}_{2}$ reduction by photocatalysis, photocatalytic self-cleaning, and hydrogen production by the splitting of water [6].

${\mathrm{TiO}}_{2}$ is one of the most used and investigated materials for photocatalysis. It has a high oxidation efficiency, is nontoxic, has a high photostability, is chemically inert, and is environmentally friendly. Furthermore, Ti is widely available in the Earth’s crust, resulting in a low cost of ${\mathrm{TiO}}_{2}$ [7]. Commercial ${\mathrm{TiO}}_{2}$ is widely available in powder form. Depending on the application, this powder can be modified to meet specific requirements, such as high thermal stability, high photocatalytic activity, etc. Upon UV illumination of ${\mathrm{TiO}}_{2}$, electron–hole pairs are created. Reaction of the latter with the surrounding molecules, can result in the creation of radicals, which are able to decompose organic compounds. The band gap of the most common phases of ${\mathrm{TiO}}_{2}$, anatase and rutile, are 3.2 eV and 3.0 eV, respectively. In [8], a scheme of the valence band maximum and the conduction band minimum for different photocatalysts is shown. Since ${\mathrm{TiO}}_{2}$ can only absorb UV light, which accounts for ca. 4% of the solar spectrum, the activities of ${\mathrm{TiO}}_{2}$ are usually limited due to low solar energy utilization. For dedicated photocatalytic reactors using artificial light, UV light sources with a photon energy larger than that of ${\mathrm{TiO}}_{2}$ are readily available, and both mercury discharge (‘blacklight’) lamps or LEDs—both emitting at 365 nm—are typically used. In addition, the fast charge recombination also often leads to low photocatalytic performance of ${\mathrm{TiO}}_{2}$. These shortcomings could be enhanced by doping ${\mathrm{TiO}}_{2}$ with metal and nonmetal elements.

In this research, the photocatalytic activity of ${\mathrm{TiO}}_{2}$ nanopowders from different suppliers is compared. In photocatalysis for air purification, it is a major challenge to immobilize the photocatalyst in such a way that it is not dragged away by the gas stream, especially in high flow velocities, such as exhausts, fume hoods, and ventilation systems. At the same time, the immobilized photocatalytic particles should remain in contact with the atmosphere and retain their large surface area. In this work, the powders are fixed onto Al substrates. The obtained thick powder layers enable the comparison between the different powders. From this comparison, a single type of ${\mathrm{TiO}}_{2}$ powder is selected for further immobilization research, since the former layers are easily damaged. Different polymers are used for immobilization onto Al substrates, thus creating polymer/${\mathrm{TiO}}_{2}$ hybrid layers. Additionally, sol–gel ${\mathrm{TiO}}_{2}$/${\mathrm{TiO}}_{2}$ powder hybrid layers are prepared to immobilize the nanopowder. The physical properties and photocatalytic degradation of all samples are measured and compared.

## 2. Materials and Methods

### 2.1. Materials

Table 1 provides an overview of the samples on Al substrates prepared in this research; the type of ${\mathrm{TiO}}_{2}$ powder, the deposition method, and the sample names are mentioned.

Eight different ${\mathrm{TiO}}_{2}$ powders were used for comparison: Aeroxide P25, Aeroxide P90, Aeroxide PF2, and Aeroxide T805 from Evonik Industries; Kronoclean 7000 and Kronoclean 7050 from Kronos; and CristalACTiV PC105 and CristalACTiV PC500 from Cristal—hereafter referred to as P25, P90, PF2, T805, K7000, K7050, PC105, and PC500, respectively. These powders were used as received, without any further purification or processing.

After the analysis of the photocatalytic activity of the ${\mathrm{TiO}}_{2}$ powders, P25 was selected for investigating other immobilization methods, as explained later in the text. P25 ${\mathrm{TiO}}_{2}$ was mixed with three types of polymer solutions: polyethylene glycol (PEG M.W. 20,000), polyvinylpyrrolidone (PVP M.W. 8000), and poly(methyl methacrylate) (PMMA M.W. 35,000). For dissolution of the polymers, acetone (≥99.5% for PMMA and PEG) or ethanol (≥99.8% for PVP) was used.

This P25 ${\mathrm{TiO}}_{2}$ powder was also used for a hybrid sol–gel ${\mathrm{TiO}}_{2}$/P25 ${\mathrm{TiO}}_{2}$ coating. For the preparation of this coating, 2-methoxyethanol (≥99.8%), titanium isopropoxide (TTIP) (≥97%), and distilled $H_{2}O$ were used. As a reference, sol–gel ${\mathrm{TiO}}_{2}$ samples without the addition of P25 ${\mathrm{TiO}}_{2}$ were synthesized.

For the photocatalytic activity measurements, the decomposition of absolute ethanol (≥99.9%) was studied.

### 2.2. Synthesis

For the comparison of the commercial ${\mathrm{TiO}}_{2}$ powders, samples were prepared on aluminum substrates. First, the aluminum substrate was machined to remove a square of 2 cm × 2 cm with a depth of 100 μm in the middle of the aluminum plate. Next, 0.3 g of ${\mathrm{TiO}}_{2}$ powder was added to 300 μL 1,2 propanediol [9] and mixed in a mortar until a homogeneous paste was obtained. This paste was then placed onto the aluminum substrate and spread in the depth profile of 100 μm using a razor blade, so that it was fully filled with this ${\mathrm{TiO}}_{2}$ paste. The samples were placed in an oven at 150 °C for two hours. This resulted in a thick (100 μm) layer of ${\mathrm{TiO}}_{2}$ powder, which was suitable for measurement. It should be noted, however, that these layers were prone to damage upon handling.

In order to prepare the hybrid layers, the polymers were first dissolved in acetone (for PMMA and PEG) or ethanol (for PVP). 3 g of polymer was dissolved in 120 mL solvent for an hour in an ultrasonic bath. Next, 0.5 g of P25 was added to each solution and again placed in an ultrasonic bath for 20 min. Aluminum substrates (76 mm × 26 mm) were dip-coated (DC) in these solutions using a Bungard BEL RDC21-K dip-coater, resulting in polymer/P25 ${\mathrm{TiO}}_{2}$ hybrid layers. The following settings were used on the dip-coater: a downward moving speed of 200 mm/min (*v${}_{down\_dip}$*), an upward moving speed of 60 mm/min (*v${}_{up\_dip}$*), and a time (*t${}_{down}$*) of 1 min during which the substrate was inside the solution without moving. These samples with PEG, PVP, and PMMA were named *PEG P25*, *PVP P25*, and *PMMA P25*, respectively.

For the sol–gel-coated samples, first, 0.5 g of P25 ${\mathrm{TiO}}_{2}$ powder was mixed with 24 mL of 2-methoxyethanol at 80 ${}^{{}^{\circ}}$C using a magnetic stirrer overnight. Second, flasks covered with flexible laboratory film were purged with $N_{2}$ to remove air. A total 20 mL of 2-methoxyethanol and 4.6 mL of titanium isopropoxide (TTIP) was added to these flasks. The first solution was then injected into the latter solution. Finally, 0.6 mL of $H_{2}O$ + 2 mL 2-methoxyethanol was added to the solution, which was mixed for 30 min. Aluminum substrates (76 mm × 26 mm) were coated using either dip-coating, with the same settings as mentioned above, or spray-coating (SC) of one layer, using a Harder and Steenbeck airbrush with a 0.2 mm nozzle and air pressure of 3 bar, resulting in a sol–gel ${\mathrm{TiO}}_{2}$/P25 ${\mathrm{TiO}}_{2}$ hybrid layer. These samples were named *DC sol–gel P25* and *SC sol–gel P25*, respectively. The same process was also performed to synthesize the reference sol–gel ${\mathrm{TiO}}_{2}$ samples, with the exception that no P25 ${\mathrm{TiO}}_{2}$ powder was added. These reference samples were named *DC sol–gel ref* and *SC sol–gel ref*, respectively.

### 2.3. Experimental Methods

Powder X-ray diffraction (PXRD) measurements were performed on all ${\mathrm{TiO}}_{2}$ powders in order to obtain information about the crystal structure of the samples. A Bruker D5000 *θ–2θ* diffractometer with Cu Kα radiation (λ = 0.154 nm, 40 kV, 40 mA) was used for these measurements, with 2θ ranging from 10${}^{{}^{\circ}}$–80${}^{{}^{\circ}}$, a step size of 0.02${}^{{}^{\circ}}$ and a dwell time of 1.5 s per step. The data were analyzed with EVA software from Bruker (Billerica, MA, USA). Scherrer’s equation [10] was used to estimate crystallite sizes by determining the FWHM of the XRD peaks at 25${}^{{}^{\circ}}$ and 48${}^{{}^{\circ}}$, taking into account the instrumental broadening. An average was calculated from these crystallite sizes.

Scanning electron microscopy (SEM) was performed to study the layer morphology using a FEI Quanta 200 F instrument at high vacuum. This technique was combined with energy dispersive X-ray spectroscopy (EDX) using EDAX Genesis 4000 hardware and software to identify the elements in the layers.

Diffuse reflectance measurements were performed using a Perkin Elmer Lambda 1050 UV-Vis-NIR spectrophotometer with an integrating sphere as detector. The measured diffuse reflectance spectra were converted to absorbance spectra using the Kubelka–Munk (K–M) transform [11].

The photocatalytic degradation of ethanol was measured with a stainless steel batch reactor (volume 8.75 L) connected to a Pfeiffer Vacuum GSD 301 C2 quadrupole mass spectrometer (QMS). A detailed description of this setup can be found in [12]. The samples were mounted inside this reactor on a heating stage, which was kept at 40 ${}^{\circ }$C during the measurements, to avoid temperature fluctuations by, e.g., the light source. A pumping system (rotary and turbomolecular pump, Pfeiffer Vacuum) was used for evacuating the stainless steel reactor, after which the latter was disconnected from these pumps by closing a valve. A gas mixture Ar/O${}_{2}$ (80%/20%) was introduced up to a pressure of 1050 mbar, to create a slight overpressure and avoid air leaking in. Ethanol (6 μL) was injected into the reactor chamber as the VOC, corresponding to a VOC concentration of 273 ppm. A small fan inside was responsible for circulation of the gas mixture. After stabilization during 1 h, the sample was irradiated by the light of a UV mercury high-pressure discharge lamp (100 W), which entered the reactor through a quartz window. This lamp emits UV light with dominant wavelengths 254 nm, 297 nm, 302 nm, 313 nm, 334 nm, and 365 nm, all suitable to activate ${\mathrm{TiO}}_{2}$ (band gap for anatase, 3.2 eV; for rutile, 3.0 eV) [12]. The QMS then analyzed gas samples from the reactor chamber as a function of time. For the dip-coated and spray-coated samples, two coated aluminum slides were mounted simultaneously to increase the irradiated surface.

The degradation of ethanol was observed by measuring at the mass-to-charge ratio (*m*/*z*) at 45 amu. This measured signal from the QMS was corrected for the contribution of ${\mathrm{CO}}_{2}$ to the 45-amu signal. This signal was divided by the signal from Ar (*m*/*z* = 40 amu) to correct for the decrease of the signal by the QMS, due to the continuous gas sampling from the reactor chamber. This ratio was then converted to normalized concentrations, displayed in the decomposition graphs in this work. For greater accuracy, only the parts of these graphs above 30% are considered for further analysis, indicated by a horizontal dashed line, since the contribution of ${\mathrm{CO}}_{2}$ to the 45 amu signal is still nonzero after degradation. This still enables comparison of the photocatalytic activity of the different ${\mathrm{TiO}}_{2}$ samples. In this research, the photocatalytic activity of different samples will be indicated by the normalized ethanol concentration in the reactor chamber 1 h after the start of the measurement, compared with the initial ethanol concentration. In the decomposition graphs, this is denoted by a vertical dashed line at 1 h.

## 3. Results and Discussion

### 3.1. TiO_2_ Powder Comparison

**Figure 1:**
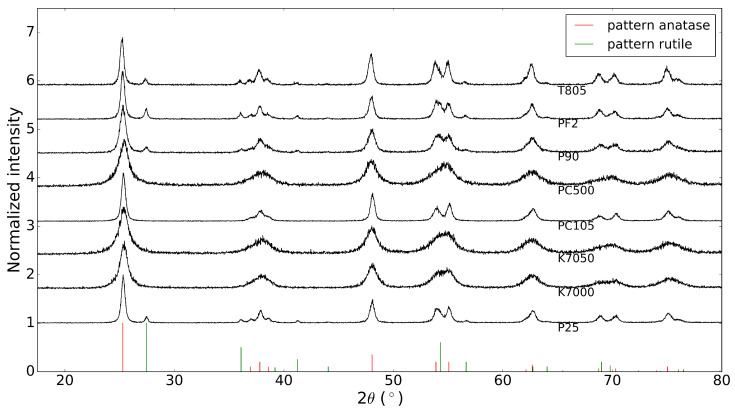
X-ray diffraction patterns of commercial ${\mathrm{TiO}}_{2}$ powders (anatase PDF number: 00-021-1272, rutile PDF number: 00-021-1276 [13]).

Before depositing the commercial ${\mathrm{TiO}}_{2}$ powders on a substrate, the X-ray diffraction patterns of these ’raw’ powders were acquired. These results are shown in Figure 1, together with the database patterns of anatase and rutile, with PDF numbers 00-021-1272 and 00-021-1276, respectively [13]. It is clear that the ${\mathrm{TiO}}_{2}$ powders from Evonik Industries indeed consist of a mixture of anatase and rutile, while the other powders are pure anatase, as stated by the manufacturers.

In Table 2, the estimation of the crystallite sizes from the Scherrer equation is shown, as well as the surface area of the powders, which is found in the product data sheets provided by the manufacturers. The Scherrer equation was applied to the XRD peaks with a 2θ value of around 25${}^{\circ }$ and 48${}^{\circ }$. As expected, larger crystallites correspond to lower surface area.

**Figure 2:**
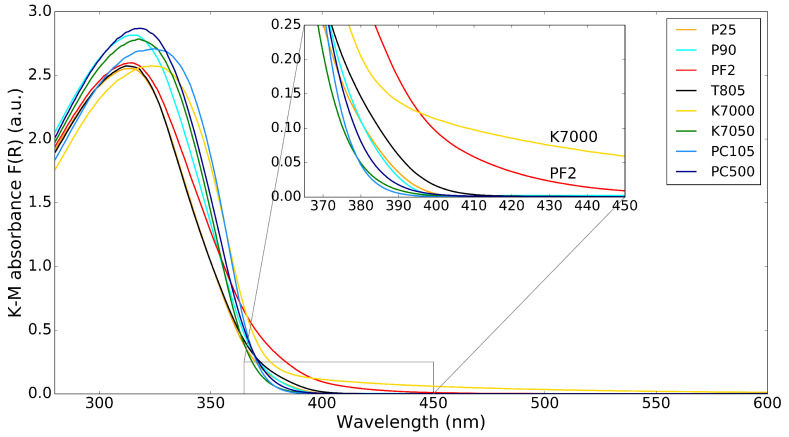
K–M absorbance spectrum for different ${\mathrm{TiO}}_{2}$ powders. The inset shows the region from 365–450 nm.

The K–M absorbance spectra of the commercial ${\mathrm{TiO}}_{2}$ powders are shown in Figure 2, with an inset showing the absorbance in the lower wavelength part of the visible spectrum (364–450 nm) to highlight the absorption of visible light by the ${\mathrm{TiO}}_{2}$ nanopowders. There are only small differences in absorbance of the different ${\mathrm{TiO}}_{2}$ powders. The most interesting differences are for the K7000 and PF2 samples, which absorb some visible light up to 450 nm, as can be seen from the inset. The reason for this is that K7000 is ${\mathrm{TiO}}_{2}$ with a carbon-modified surface, while PF2 is a mixture of ${\mathrm{TiO}}_{2}$ and iron-oxide. This visible light absorption can be beneficial for visible light photocatalysis. It is common to try to increase the sensitivity of ${\mathrm{TiO}}_{2}$ photocatalysts to visible light by doping with N or Fe [14]; Cu, Ni, Zn, or Pb [15], ${\mathrm{ZrO}}_{2}$ [16]; and V, Nb, or Ta [17]. However, it is rare to find such doped photocatalysts in commercial nanopowders.

**Figure 3:**
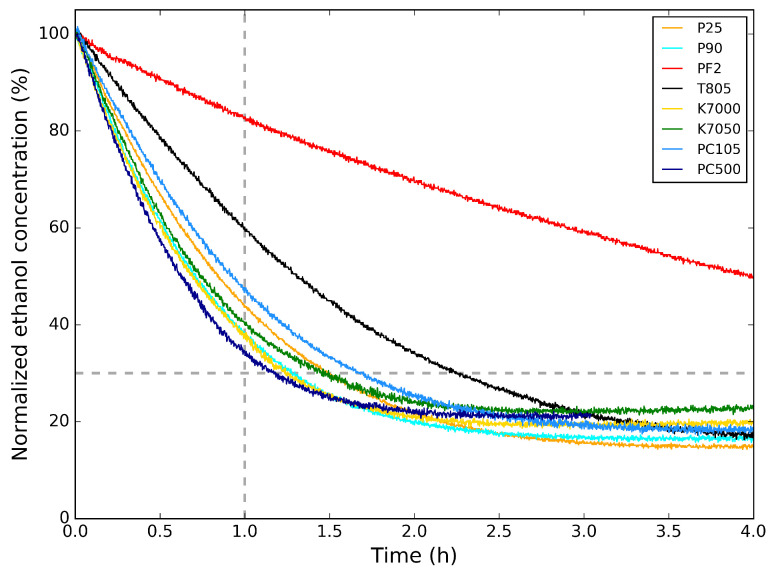
Photocatalytic degradation of ethanol for the ${\mathrm{TiO}}_{2}$ powders measured at *m*/*z* = 45 amu. Only the part of the graph above the dashed line is considered for greater accuracy, since it is an artifact of the correction (see text).

In Figure 3, the photocatalytic activity of the ${\mathrm{TiO}}_{2}$ powders for the degradation of ethanol is shown. Here, the ethanol concentration is normalized to its initial concentration. In Table 3, the relative ethanol concentration values 1 h after the start of the photocatalytic degradation are listed. This is an indication of the photocatalysis rate of the ${\mathrm{TiO}}_{2}$ samples. Comparing the photocatalytic degradation graphs and the values from Table 3 with the crystallite sizes in Table 2, it is clear that the ${\mathrm{TiO}}_{2}$ powders with smaller crystallite sizes (PC500, P90, K7000, and K7050) induce a faster photocatalytic abatement of ethanol. The photocatalytic abatement for P25 and PC105, with larger crystallite sizes, is only slightly slower, indicating a subtle difference in photocatalytic activity. T805 and PF2 are less suitable for photocatalytic abatement of ethanol, with the former mostly applied as additive for toners for copiers and printers and the latter as heat stabilizer in silicones.

For the photocatalytic degradation of ethanol, both reproducibility and repeatability were studied to estimate the absolute error for the values in Table 3. For the former, the photocatalytic activity for different samples prepared using the same synthesis method was tested, while for the latter, the photocatalytic activity of the same sample was repeated. An absolute error for the percentage of normalized ethanol concentration after 1 h of photocatalytic degradation was estimated to 5% (Table 3), mostly related to the conditions of the experimental setup.

### 3.2. Polymer/TiO_2_ and Sol–Gel/TiO_2_ Hybrid Layers

**Figure 4:**
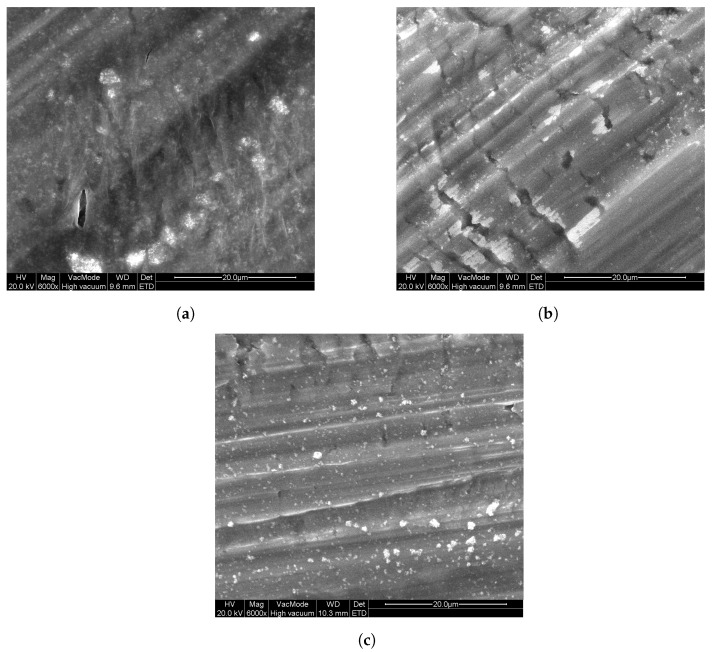
SEM images of the polymer/${\mathrm{TiO}}_{2}$ coatings on aluminum substrates: (**a**) PEG P25, (**b**) PVP P25, and (**c**) PMMA P25.

Since the abovementioned ${\mathrm{TiO}}_{2}$ powder layers are easily damaged upon handling, another deposition approach is required to form photocatalytic layers for long-term usage. However, the results from Figure 3 enables us to select a suitable ${\mathrm{TiO}}_{2}$ powder for further investigation. P25 ${\mathrm{TiO}}_{2}$ powder is selected, since it is often used as a reference ${\mathrm{TiO}}_{2}$ powder in research. Additionally, because the photocatalytic properties are similar to that of the other powders (except T805 and PF2), it is meaningful to use this photocatalyst as benchmark. Immobilization of this powder with different polymers (see Section 2) was investigated by dip-coating on Al substrates. In Figure 4, images acquired by SEM can be seen that show the surface morphology of these layers. From these, it can be seen that there are some particles present at the surface of these layers. These particles contain titanium, as confirmed by EDX measurements and are from the P25 powder. The layers seem firm with some slight cracking. The marked striations in the images are due to the large surface roughness of the aluminum substrates.

**Figure 5:**
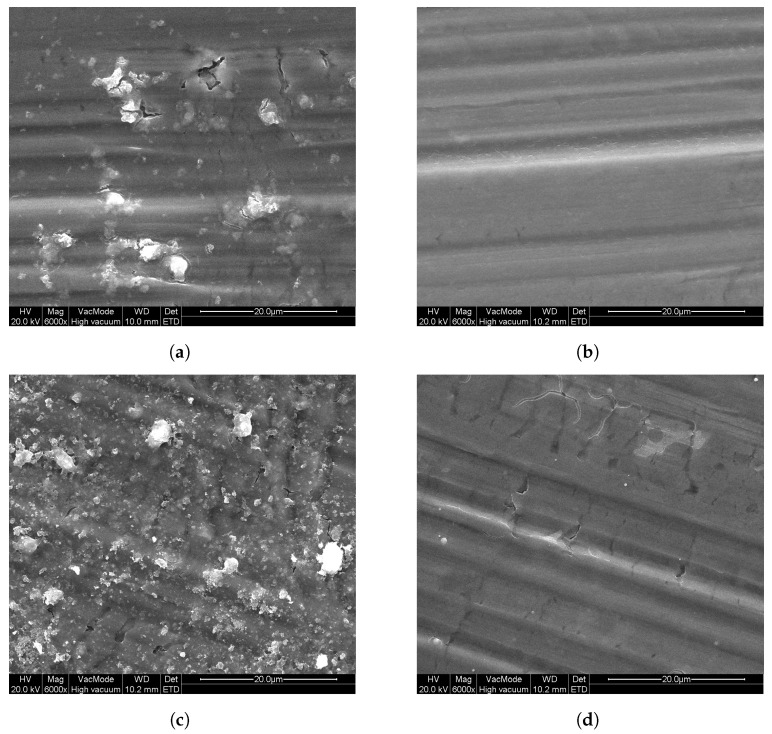
SEM images of the sol–gel ${\mathrm{TiO}}_{2}$ coatings on aluminum substrates: (**a**) DC sol–gel P25, (**b**) DC sol–gel ref, (**c**) SC sol–gel P25, and (**d**) SC sol–gel ref.

Additionally, P25 was coated onto Al substrates using a sol–gel method, as described in Section 2, by dip-coating and spray-coating. As a reference, sol–gel ${\mathrm{TiO}}_{2}$ samples were prepared without the addition of P25 ${\mathrm{TiO}}_{2}$. In Figure 5, SEM images of these samples can be seen that show the surface morphology of these layers. From these images, it is clear that all sol–gel-prepared samples consist of firm layers onto the Al substrates, with only slight cracking. For the DC sol–gel P25 sample, in the homogeneous layer, particles can be seen in Figure 5a (lighter parts), of which EDX measurements confirmed that these particles contain titanium. For the DC sol–gel reference (Figure 5b) sample, these particles are absent. Looking at the spray-coated samples, the surface of SC sol–gel P25 in Figure 5c is similar to DC sol–gel P25, but with a higher concentration of titanium-containing particles at the surface, as was confirmed by EDX measurements. These particles are the added P25 ${\mathrm{TiO}}_{2}$. Comparing SC sol–gel P25 to SC sol–gel reference (Figure 5d), the surface of the reference sample does not contain P25.

**Figure 6:**
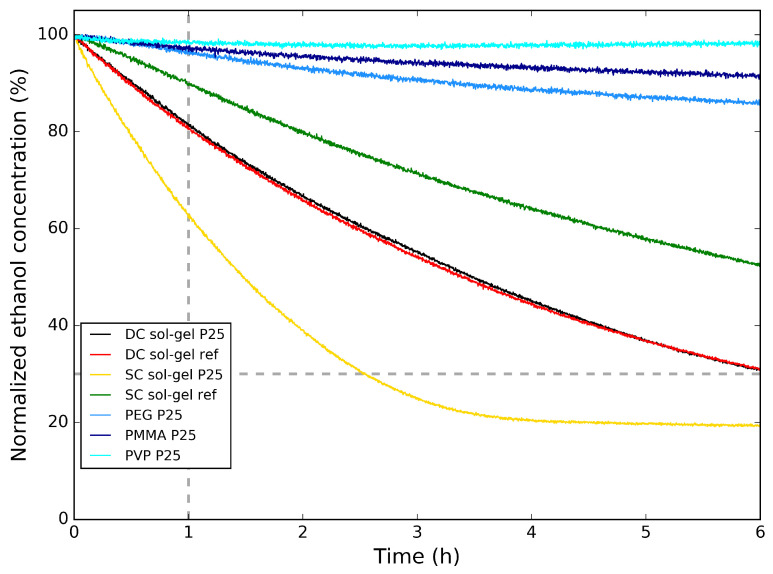
Normalized ethanol concentration vs. time for the polymer/P25 ${\mathrm{TiO}}_{2}$ samples on Al and the sol–gel ${\mathrm{TiO}}_{2}$ reference and the sol–gel ${\mathrm{TiO}}_{2}$/P25 ${\mathrm{TiO}}_{2}$ samples on Al during the photocatalytic degradation tests of ethanol.

In Figure 6, the photocatalytic degradation of ethanol for the polymer/P25 ${\mathrm{TiO}}_{2}$ hybrid samples on aluminum substrate are compared to the sol–gel ${\mathrm{TiO}}_{2}$ reference and the sol–gel ${\mathrm{TiO}}_{2}$/P25 ${\mathrm{TiO}}_{2}$ hybrid samples on aluminum substrate. Here, the normalized ethanol concentration is shown as a function of time. First, the polymer/P25 ${\mathrm{TiO}}_{2}$ samples are compared. Here, it is clear that the type of polymer has an influence on the rate of photocatalytic degradation. The ${\mathrm{TiO}}_{2}$ particles are embedded in the polymer and the polymers are not porous, resulting in a low amount of ${\mathrm{TiO}}_{2}$ directly in contact with the surrounding atmosphere, decreasing the reaction surface. This explains why, compared with the dip-coated sol–gel ${\mathrm{TiO}}_{2}$ reference and the sol–gel ${\mathrm{TiO}}_{2}$/P25 ${\mathrm{TiO}}_{2}$ samples, the photocatalytic degradation of ethanol is much slower—also indicated in Table 3—with between 96% and 98% of the initial ethanol concentration remaining in the reactor chamber after 1 h for the polymer/P25 ${\mathrm{TiO}}_{2}$ samples, while for the dip-coated sol–gel ${\mathrm{TiO}}_{2}$ samples, there is 81% of the initial ethanol concentration remaining in the reactor chamber after 1 h. From this improvement, it can be concluded that samples prepared by the sol–gel ${\mathrm{TiO}}_{2}$ synthesis are more suited for photocatalytic degradation of VOC. The results from the sol–gel ${\mathrm{TiO}}_{2}$ reference and the sol–gel ${\mathrm{TiO}}_{2}$/P25 ${\mathrm{TiO}}_{2}$ are almost identical.

The photocatalytic activity can still be improved when compared with the photocatalytic activity of the bulk powder samples from Figure 3. Since the sol–gel ${\mathrm{TiO}}_{2}$ samples show the best photocatalytic activity, this sample is also prepared onto Al substrate using spray-coating, both with and without addition of P25 ${\mathrm{TiO}}_{2}$ in the synthesis. The photocatalytic degradation of ethanol from these samples is also shown in Figure 6. It can be seen that the spray-coated sol–gel ${\mathrm{TiO}}_{2}$ reference samples have a slower photocatalytic degradation than the dip-coated sol–gel ${\mathrm{TiO}}_{2}$ samples, with still 90% of the initial ethanol concentration remaining in the reactor chamber after 1 h. When adding P25 ${\mathrm{TiO}}_{2}$ to the spray-coated solution, this improves to 63%, also listed in Table 3. This means that for spray-coating, the addition of commercial P25 ${\mathrm{TiO}}_{2}$ powder enhances the photocatalytic activity of the coating. Comparing the spray-coated sol–gel P25 with the dip-coated sol–gel P25, it is clear that the photocatalytic activity is greatly enhanced, which is expected looking at the SEM images (Figure 5a,c, respectively) from the visibly higher concentration of P25 at the surface for the former.

It can be concluded that synthesizing a sol–gel ${\mathrm{TiO}}_{2}$/P25 ${\mathrm{TiO}}_{2}$ hybrid layer using a spray-coating technique results in coatings that possess a high photocatalytic activity for degradation of ethanol. Still, the rate of photocatalytic activity is slightly lower than that of bulk powder. This spray-coating method could still be improved by varying the spray-coating parameters (nozzle size, spraying time and distance, etc.).

## 4. Conclusions

Photocatalytically active ${\mathrm{TiO}}_{2}$ powder is widely available and can be easily obtained from different suppliers. In this work, different ${\mathrm{TiO}}_{2}$ powders from Evonik Industries, Kronos, and Cristal were investigated and compared with each other. Applying a first immobilization technique, by spreading a paste of 1,2 propanediol and ${\mathrm{TiO}}_{2}$ powder onto aluminum slides, resulted in ∼100 μm-thick bulk powder layers. Among the ${\mathrm{TiO}}_{2}$ powders, P25 was selected due to its photocatalytic performance and its regular role as a reference photocatalytic material. In order to prevent damage from gas streams, hybrid immobilization techniques were developed using this particular powder.

Polymer/P25 ${\mathrm{TiO}}_{2}$ hybrid layers on Al were prepared by dip-coating these substrates. Limited photocatalytic degradation of ethanol was obtained, most probably due to the limited amount of ${\mathrm{TiO}}_{2}$ on the surface of these polymer layers and the resulting low effective area available for photocatalytic reaction. Sol–gel ${\mathrm{TiO}}_{2}$/P25 ${\mathrm{TiO}}_{2}$ hybrid layers on Al were also prepared by dip-coating, as well as a reference without addition of P25 ${\mathrm{TiO}}_{2}$. The photocatalytic performance of these samples was increased compared with that of the polymer/P25 ${\mathrm{TiO}}_{2}$ samples. The photocatalytic degradation of ethanol of the sol–gel ${\mathrm{TiO}}_{2}$/P25 ${\mathrm{TiO}}_{2}$ hybrid layer was enhanced by using a spray-coating technique, due to the higher P25 concentration at the surface, visible from the SEM images. The photocatalytic activity of the latter was only slightly lower than that of the bulk powder layers. Thus, the commercial P25 ${\mathrm{TiO}}_{2}$ was successfully immobilized while only slightly decreasing the photocatalytic efficiency of the material. The spray-coated sol–gel ${\mathrm{TiO}}_{2}$ reference sample, without commercial P25 ${\mathrm{TiO}}_{2}$ showed a much slower photocatalytic degradation. The spray-coating method could still be optimized—by changing, e.g., the nozzle size, spraying time, and distance—and applied to the other commercial ${\mathrm{TiO}}_{2}$ nanopowders. The presented methods offer a way to obtain stable coatings with high photocatalytic activity, which can be used in purification of gas streams with high flow rates.

## Figures and Tables

**Table 1:** Overview of the prepared samples on Al substrates.

	Bulk	Polymer (PEG, PVP or PMMA)	Sol–Gel ${\mathrm{TiO}}_{2}$	Sol–Gel ${\mathrm{TiO}}_{2}$
${\mathrm{TiO}}_{2}$ powder added	P25; P90; PF2; T805; K7000; K7050; PC105; PC500 *	P25	P25	-
deposition method	spreading paste with razor blade	dip-coating	dip-coating (DC) or spray-coating (SC)	dip-coating or spray-coating
sample name	P25; P90; PF2; T805; K7000; K7050; PC105; PC500	PEG P25;PVP P25;PMMA P25	DC sol–gel P25;SC sol–gel P25	DC sol–gel ref; SC sol–gel ref

* See references in the text.

**Table 2:** Estimated crystal size and the listed surface area for the ${\mathrm{TiO}}_{2}$ samples.

	P25	P90	PF2	T805	K7000	K7050	PC105	PC500
Estimated crystal size (nm)	32	17	30	31	11	9	33	10
Surface area (m${}^{2}$/g)	35–65	70–110	45–70	35–55	>225	>225	∼90	∼350

**Table 3:** Percentage of normalized ethanol concentration, relative to the initial ethanol concentration, after 1 h of photocatalytic degradation for the different ${\mathrm{TiO}}_{2}$ samples discussed in this work. An absolute error for these values was estimated to 5%.

Sample Name	$C_{\mathrm{EtOH}}/{C_{0}}_{\mathrm{EtOH}}$ (1 h) (%)	Sample Name	$C_{\mathrm{EtOH}}/{C_{0}}_{\mathrm{EtOH}}$ (1 h) (%)
P25	44	PEG P25	96
P90	38	PVP P25	98
PF2	82	PMMA P25	98
T805	60	DC sol–gel ref	81
K7000	38	SC sol–gel ref	90
K7050	40	DC sol–gel P25	81
PC105	48	SC sol–gel P25	63
PC500	34		

## Data Availability

The data presented in this study are available on request from the corresponding author.
